# High-Quality Conjugated Polymers Achieving Ultra-Trace Detection of Cr_2_O_7_^2−^ in Agricultural Products

**DOI:** 10.3390/molecules27134294

**Published:** 2022-07-04

**Authors:** Hui Li, Fei Li, Fang Liu, Xiao Chen, Wenyuan Xu, Liang Shen, Jingkun Xu, Rui Yang, Ge Zhang

**Affiliations:** 1 School of Pharmacy, Jiangxi Science and Technology Normal University, Nanchang 330013, China; lh11123210823@163.com (H.L.); lifei0262@163.com (F.L.); liufang1997@126.com (F.L.); 2 School of Chemistry and Chemical Engineering, Jiangxi Science and Technology Normal University, Nanchang 330013, China; cx1351420@163.com (X.C.); x13698018030@163.com (W.X.); liangshen@vip.163.com (L.S.); 3 Jiangxi Key Lab of Flexible Electronics, Flexible Electronics Innovation Institute, Jiangxi Science and Technology Normal University, Nanchang 330013, China; 4 College of Chemistry and Molecular Engineering, Qingdao University of Science and Technology, Qingdao 266042, China; 5 School of Electronics and Information Engineering, Changshu Institute of Technology, Changshu 215500, China

**Keywords:** Cr_2_O_7_^2−^, design strategy, conjugated polymer, ultra-trace detection, agricultural products

## Abstract

In view of that conjugated polymers (CPs) are an attractive option for constructing high-sensitive Cr_2_O_7_^2−^ sensors but suffer from lacking a general design strategy, we first proposed a rational structure design of CPs to tailor their sensing properties while validating the structure-to-performance correlation. Short side chains decorated with N and O atoms as recognition groups were instructed into fluorene to obtain monomers Fmoc-Ala-OH and Fmoc-Thr-OH. Additionally, their polymers **P(Fmoc-Ala-OH)** and **P(Fmoc-Thr-OH)** were obtained through electrochemical polymerization. **P(Fmoc-Ala-OH)** and **P(Fmoc-Thr-OH)** with high polymerization degrees have an excellent selectivity towards Cr_2_O_7_^2−^ in comparison to other cations and anions. Additionally, their limit of detection could achieve 1.98 fM and 3.72 fM, respectively. Especially, they could realize the trace detection of Cr_2_O_7_^2−^ in agricultural products (red bean, black bean, and millet). All these results indicate that short side chains decorated with N and O atoms functionalizing polyfluorene enables the ultra-trace detection of Cr_2_O_7_^2−^. Additionally, the design strategy will spark new ideas for the construction of highly selective and sensitive Cr_2_O_7_^2−^ sensors.

## 1. Introduction

Environmental pollution jeopardizes people’s health even at a very low concentration due to its high toxicity, so it is receiving more and more attention [1]. If we want to formulate a reasonable and feasible treatment plan, it is necessarily to monitor these pollutions quickly, easily, and accurately. Recently, fluorescence analysis methods have been developing rapidly, and these enable high selectivity and sensitivity, fast detection, in situ analysis, and low cost [2]. Among various fluorescent materials, conjugated polymers (CPs) are comparatively dominant because they could introduce recognition groups and have a unique “molecular wire effect” [3,4,5], which greatly improves the selectivity and sensitivity of fluorescent sensors [6,7,8,9]. In the past decades, CPs-based fluorescent sensors have been the research hotpot.

Chromium (Cr) is one necessary element in industry, but the impact of hexavalent chromium (Cr(VI)) on human health should not be underestimated [10,11,12]. Once its content exceeds the standard in water (existing in the form of Cr_2_O_7_^2^^−^), it will be enriched in the crops and passed to the human body through the food chain, which will cause various diseases [13,14,15,16,17,18,19]. Up to now, Cr_2_O_7_^2^^−^ detection materials mainly contain inorganic materials (quantum dots [20,21,22] and carbon dots [23,24,25]), organic materials (organic molecules [26,27] and CPs [28]), and organic–inorganic hybrid materials (metal-organic frameworks [29,30] and nanoclusters). Generally, inorganic materials recognize Cr_2_O_7_^2^^−^ by inner filtering effect (IFE) and organic materials used recognition groups through hydrogen bonds or coordination bonds, while organic–inorganic hybrid materials often rely on the synergetic effect of the aforementioned recognition mechanism of inorganic and organic materials. For Cr_2_O_7_^2^^−^, which are difficult to detect at low concentrations, CPs show the advantages of trace detection. Unfortunately, few efforts have been devoted to the development of this kind of sensor based on CPs, which may be the difficulties of design and preparation. Our group have been committed to the design and development of new CPs and apply them in fluorescent sensing fields [12,13,14,15]. Indeed, CPs exhibit supersensitivity in detecting targets. In our previous work, we have prepared several Cr_2_O_7_^2^^−^ sensors based on CPs, but only two sensors could achieve ultra-trace detection. Thus, it remains a challenge to design CP-based sensors with high performance and validate a general design strategy.

In this work, we aim to rationally design the structure of CPs to tailor their sensing properties while validating the structure-to-performance correlation. Fluorene, an excellent blue emitter, was selected as the fluorophore. The steric hindrance of side chain will hinder the polymerization of fluorene and decrease the polymerization degree, which is closely related to the detection sensitivity. So, the side chains were customized with desired N and O atoms as Cr_2_O_7_^2^^−^ recognition group, and their chain length was kept as short as possible. Fluorene modified by two acid groups were synthesized, and their polymers (**P(Fmoc-Ala-OH)** and **P(Fmoc-Thr-OH)**) were prepared through electrochemical polymerization. A series of experiments show that fluorescent sensors based on **P(Fmoc-Ala-OH)** and **P(Fmoc-Thr-OH)** could achieve the trace detection of Cr_2_O_7_^2^^−^ in agricultural products.

## 2. Results and Discussion

### 2.1. Electropolymerization of Monomers Fmoc-Ala-OH and Fmoc-Thr-OH

**Figure 1:**
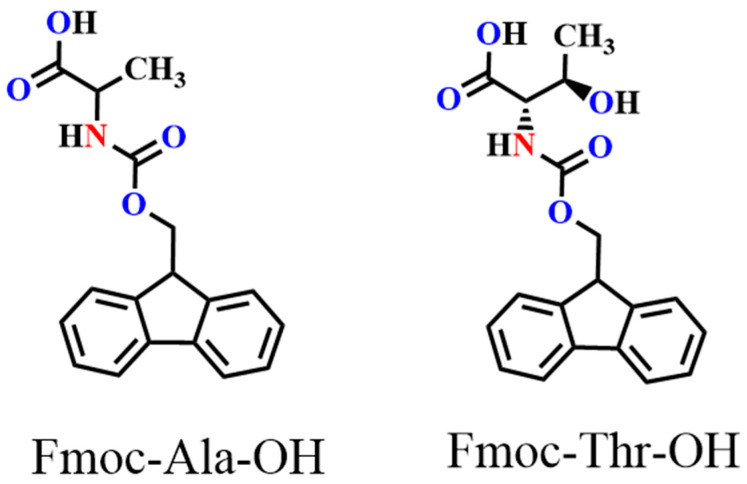
Structure of monomers Fmoc-Ala-OH and Fmoc-Thr-OH.

**Figure 2:**
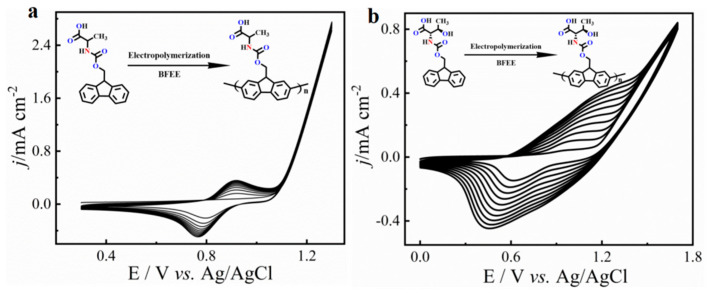
Multicycle CVs of monomer Fmoc-Ala-OH (**a**) and Fmoc-Thr-OH (**b**) in the BFEE system. Potential scan rate of 100 mV s^−1^.

In this work, we chose an acid group decorated with N and O atoms as the functional side chains. To keep the side chain as short as possible, every side chain contained only one -NH_2_ and one -COOH unit, and two fluorene derivatives were obtained as monomers (Figure 1). Two monomers could easily dissolve in common solvents, such as tetrahydrofuran (THF), *N*,*N*-Dimethylformamide (DMF), and dichloromethane (DCM). However, in neutral solvents, they could not polymerize any supporting electrolytes including Bu_4_NPF_6_, Bu_4_NBF_4_, and Bu_4_NClO_4_ (Table S1). Delightfully, they could easily polymerize in a pure boron trifluoride ethyl ether (BFEE) system without adding external supporting electrolyte because BFEE can lower the polymerization potential and promote the polymerization of fused ring compounds. As shown in Figure 2, as the number of scan cycles increased, the current density of a pair of reversible redox peaks in cyclic voltammograms (CVs) increased, indicating that monomers Fmoc-Ala-OH and Fmoc-Thr-OH can be electropolymerized in BFEE. By analysing the ^1^H NMR spectra (Figures S1 and S2), it was found that two molecules were polymerized at the position 2,7, which was the same as other fluorene derivatives [13,14,15,31]. From GPC results, polymers **P(Fmoc-Ala-OH)** and **P(Fmoc-Thr-OH)** contained 72 and 41 repeat units, respectively. In other words, the two polymers have high polymerization degrees. Combined with other polyfluorene reported by our groups [12,13,14,15], the shorter side chain groups will reduce the polymerization hindrance of fluorene. Importantly, high polymerization degrees are beneficial to the detection sensitivity in application.

### 2.2. Selective and Competitive Testing of Polymers P(Fmoc-Ala-OH) and P(Fmoc-Thr-OH)

**Figure 3:**
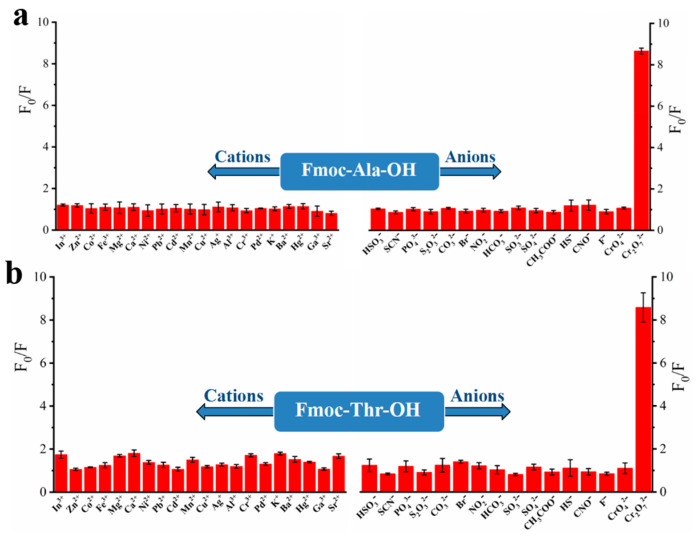
Fluorescence quenching of monomer Fmoc-Ala-OH (**a**) and Fmoc-Thr-OH (**b**) to various cations/anions.

**Figure 4:**
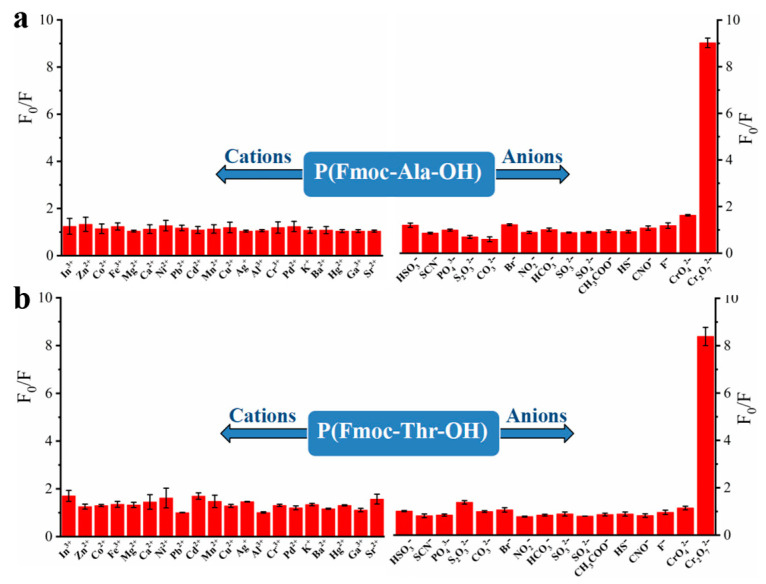
Fluorescence quenching of **P(Fmoc-Ala-OH)** (**a**) and **P(Fmoc-Thr-OH)** (**b**) to various cations/anions.

**Figure 5:**
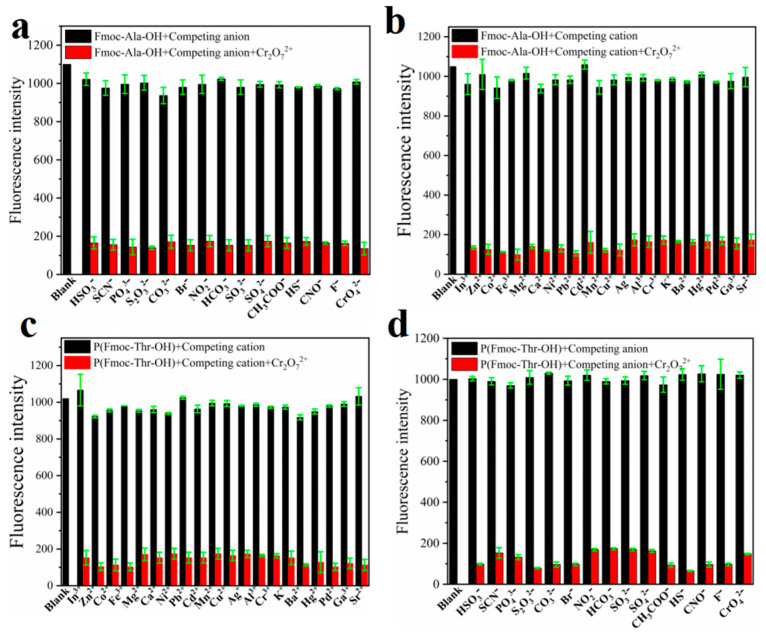
Fluorescence intensity of **P(Fmoc-Ala-OH)** (**a**,**b**) and **P(Fmoc-Thr-OH)** (**c**,**d**) in the mixed DMSO/EtOH (*v*/*v* = 1:800) containing various ions. The black bars represent the addition of the competing ions to a solution of **P(Fmoc-Ala-OH)** and **P(Fmoc-Thr-OH)**. The red bars represent the change of the emission that occurs upon the subsequent addition of Cr_2_O_7_^2−^ to the above solution.

To explore the recognition of acid groups in fluorene, we first studied the selectivity of two monomers to common anions and cations. From Figure 3, we can see that only Cr_2_O_7_^2^^−^ could quench the fluorescence of monomers Fmoc-Ala-OH and Fmoc-Thr-OH, which indicated that they have high selectivity to Cr_2_O_7_^2^^−^. We speculate that the N and O atoms may interact with Cr_2_O_7_^2^^−^ [32], which caused the aggregate of fluorene and resulted in fluorescence quenching [33]. Then, we studied the selectivity of their corresponding polymers. As shown in Figure 4, all their polymers **P(Fmoc-Ala-OH)** and **P(Fmoc-Thr-OH)** also exhibited the good selectivity for Cr_2_O_7_^2^^−^, which were not interfered by anions and cations (Figure 5).

### 2.3. Sensitivity Test of P(Fmoc-Ala-OH) and P(Fmoc-Thr-OH)

**Figure 6:**
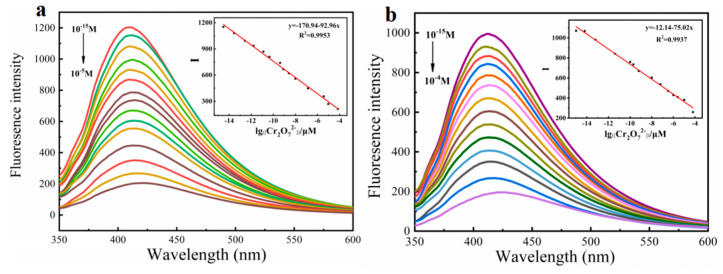
Fluorescence emission spectra of **P(Fmoc-Ala-OH)** (**a**) 2.5 μM and **P(Fmoc-Thr-OH)** (**b**) 4.2 μM toward Cr_2_O_7_^2−^ with different concentrations in DMSO-EtOH, respectively. Inset: Linear plots of their fluorescence intensity against Cr_2_O_7_^2−^ concentration. (Ex = 335 nm, 350 nm).

Based on the above results, both monomers and polymers showed specific recognition to Cr_2_O_7_^2−^, so we further explore their sensitivity. Additionally, the linear relationship between fluorescence intensity and Cr_2_O_7_^2−^ concentration was studied. As shown in Figure S1, monomers Fmoc-Ala-OH and Fmoc-Thr-OH have sensitivity to Cr_2_O_7_^2−^ in nM level, while their limits of detection (LODs) were 0.11 nM and 0.27 nM, respectively. When they were prepared into polymers, their detection sensitivity was greatly improved and reached upto fM, and their LODs achieved 1.98 fM and 3.72 fM, respectively (Figure 6). This verifies that the molecular wire effect of polymer can greatly improve the sensitivity of the detection of Cr_2_O_7_^2−^, which further indicates that this type of acid-functionalized polyfluorene fluorescence material has the ability of ultra-trace detection of Cr_2_O_7_^2−^. Compared with other Cr_2_O_7_^2−^ sensors reported, **P(Fmoc-Ala-OH)** and **P(Fmoc-Thr-OH)** showed the lowest LOD, which is owed to the delicate design of side chains and the preparation of high-quality polymers.

### 2.4. Application

To explore the feasibility of sensors **P(Fmoc-Ala-OH)** and **P(Fmoc-Thr-OH)** applied to real samples, we detected Cr_2_O_7_^2−^ in red bean, black bean, and millet samples by standard addition method [34]. As shown in Table 1 and Table 2, **P(Fmoc-Ala-OH)** and **P(Fmoc-Thr-OH)** were not significantly quenched by each agricultural products sample, indicating that these agricultural product samples may not contain Cr_2_O_7_^2−^. We then added the standard concentration of Cr_2_O_7_^2−^ to three agricultural products samples containing **P(Fmoc-Ala-OH)** and **P(Fmoc-Thr-OH)** and found that the fluorescence intensity of the samples was quenched. In actual samples, two fluorescence sensors have good detection results, and the recovery rates are (94.0–103.0%) and (91.0–101.5%), respectively, indicating that the two fluorescent sensors **P(Fmoc-Ala-OH)** and **P(Fmoc-Thr-OH)** are able to detect Cr_2_O_7_^2−^ in agricultural product samples. Based on the above results, we believe that this type of amino-acid-functionalized polyfluorene fluorescent sensor can be applied to the detection of Cr_2_O_7_^2−^ in real agricultural products.

## 3. Materials and Methods

### 3.1. Materials and Instruments

The materials and instruments used in this work, and the corresponding characterization of monomer Fmoc-Ala-OH and Fmoc-Thr-OH, are listed in the Supplementary Information.

Fmoc-Ala-OH (98%, Aladdin, Shanghai, China), Fmoc-Thr-OH (98%, Aladdin), tetrahydrofuran (THF, 99%, Aladdin), *N*,*N*-Dimethylformamide (DMF, 99%, Aladdin), dichloromethane (DCM, 99.99%, Aladdin), boron trifluoride diethyl etherate (BFEE, 98%, Aladdin) were used directly. Twice distilled water was used throughout all experiments. The aqueous solution of Sn^2+^ was prepared from its chloride salt; the aqueous solution of Ag^+^ was prepared from its perchloric acid salt; aqueous solutions of Sr^2+^, Ga^3+^, Pd^2+^, Hg^2+^, Ba^2+^, K^+^, Cr^3+^, Al^3+^, Cu^2+^, Mn^2+^, Cd^2+^, Pb^2+^, Ni^2+^, Ca^2+^, Mg^2+^, Fe^2+^, Fe^3+^, Co^2+^, Zn^2+^, and In^3+^ were prepared from their nitrate salts. The aqueous solution of Cr_2_O_7_^2−^ was prepared from its kalium salt; aqueous solutions of F^−^, CNO^−^, HS^−^, CH_3_COO^−^, SO_4_^2−^, SO_3_^2−^, HCO_3_^2−^, NO_2_^−^, Br^−^, CO_3_^2−^, S_2_O_3_^2−^, PO_4_^3−^, SCN^−^, and HSO_3_^−^ were prepared from their sodium salts. Sodium dihydrogen phosphate (NaH_2_PO_4_), disodium hydrogen phosphate (Na_2_HPO_4_), hydrochloric acid, and aqueous ammonia were purchased from Tianjin Damao Chemical Plant (Tianjin, China).

Electrochemical polymerization was performed using a Versa Stat 3 electrochemical workstation (EG&G Princeton Applied Research, Shanghai, China) under computer control. The GPC determination of P1 and P2 was carried out using American Waters 1525 gel chromatography (Waters, MA, USA; chromatographic column: Agilent PLgel 5um MIXED-C, manufactured by GB, Palo Alto, CA, USA) and the mobile phase was DMF. Absorption spectra were obtained from an Agilent 8454 UV-vis spectrophotometer (Agilent, Palo Alto, CA, USA). All fluorescent experiments were studied on a Hitachi F-4600 fluorospectrophotometer (Hitachi, Tokyo, Japan) with excitation/emission slit set at 5 nm.

### 3.2. Electrosynthesis of P(Fmoc-Ala-OH) and P(Fmoc-Thr-OH)

**P(Fmoc-Ala-OH)** and **P(Fmoc-Thr-OH)** were prepared in boron trifluoride ethyl ether (BFEE) system by direct anodization of monomer Fmoc-Ala-OH and Fmoc-Thr-OH, respectively. Electrochemical polymerization was accomplished using a classic one-chamber three-electrode system. The reference electrode was Ag/AgCl, and the working and counter electrodes were ITO. The electrochemical polymerization was carried out in the BFEE system, and the polymers **P(Fmoc-Ala-OH)** and **P(Fmoc-Thr-OH)** were obtained at voltages of 1.2 V and 1.38 V, respectively. The obtained polymer films were first rinsed by anhydrous ether and then dried under vacuum at 65 °C for 24 h. Molecular weight tests of **P(Fmoc-Ala-OH)** and **P(Fmoc-Thr-OH)** were tested used Gel Permeation Chromatography (GPC) and DMF was as mobile phase. P(Fmoc-Ala-OH): M_w_ = 45,202, M_n_ = 22,431, PDI = 2.01; P(Fmoc-Thr-OH): M_w_ = 22,920, M_n_ = 13,820, PDI = 1.65.

### 3.3. Detection of Cr_2_O_7_^2^^−^

**P(Fmoc-Ala-OH)** (2.5 × 10^−3^ M) solution and **P(Fmoc-Thr-OH)** (4.2 × 10^−3^ M) solution were prepared by dimethyl sulfoxide (DMSO). Selective, competitive, and sensitive experiments of two polymers were performed in DMSO-EtOH (*v*/*v* = 1:800) system.

### 3.4. Preparation of Agricultural Products Samples

The red bean, black bean, and millet were purchased from supermarkets. To evaluate the applicability of **P(Fmoc-Ala-OH)** and **P(Fmoc-Thr-OH)** in real samples, we carried out experiments using standard addition methods. After adding different concentrations of Cr_2_O_7_^2^^−^ to real samples, **P(Fmoc-Ala-OH)** and **P(Fmoc-Thr-OH)** were used to detect these samples with or without Cr_2_O_7_^2^^−^.

## 4. Conclusions

In conclusion, we proposed a design strategy to construct Cr_2_O_7_^2−^ sensors with high selectivity and sensitivity. Two fluorene derivatives with N and O atoms in short side chains were easily polymerize in BFEE system. Thanks to the N and O atoms, two monomers (Fmoc-Ala-OH and Fmoc-Thr-OH) and their polymers (P(Fmoc-Ala-OH) and P(Fmoc-Thr-OH)) could specific recognize Cr_2_O_7_^2−^ without avoiding the interference of common cations and anions. Additionally, two polymers showed high sensitivity to Cr_2_O_7_^2−^ and their LODs achieved fM. In particular, P(Fmoc-Ala-OH) and P(Fmoc-Thr-OH) realized the trace detection of Cr_2_O_7_^2−^ in agricultural products. All these results showed that short side chains with N and O atoms functionalized conjugated polymer chains could enable the ultra-trace detection of Cr_2_O_7_^2−^.

## Figures and Tables

**Table 1:** Determination of Cr_2_O_7_^2^^−^ in agricultural products samples solution using **P(Fmoc-Ala-OH)**.

Samples	Cr_2_O_7_^2−^ Spiked (M)	Cr_2_O_7_^2−^ Found ( $\bar{x}$ ± SD) (M)	Recovery (%)	RSD (%)
red bean	-	-	-	-
	2.00 × 10^−9^	(1.91 ± 0.03) × 10^−9^	95.5	1.57
	4.00 × 10^−7^	(4.03 ± 0.01) × 10^−7^	100.7	0.24
	1.00 × 10^−5^	(0.94 ± 0.02) × 10^−5^	94.0	2.1
black bean	-	-	-	-
	2.00 × 10^−9^	(2.02 ± 0.02) × 10^−9^	101.0	0.9
	4.00 × 10^−7^	(4.05 ± 0.06) × 10^−7^	101.2	1.4
	1.00 × 10^−5^	(1.03 ± 0.02) × 10^−5^	103.0	1.9
millet	-	-	-	-
	2.00 × 10^−9^	(2.01 ± 0.01) × 10^−9^	100.5	0.5
	4.00 × 10^−7^	(4.06 ± 0.03) × 10^−7^	101.5	0.73
	1.00 × 10^−5^	(0.98 ± 0.01) × 10^−5^	98.0	1.01

**Table 2:** Determination of Cr_2_O_7_^2^^−^ in agricultural product samples solution using **P(Fmoc-Thr-OH)**.

Samples	Cr_2_O_7_^2−^ Spiked (M)	Cr_2_O_7_^2−^ Found ( $\bar{x}$ ± SD) (M)	Recovery (%)	RSD (%)
red bean	-	-	-	-
	2.00 × 10^−9^	(1.91 ± 0.03) × 10^−9^	95.5	1.5
	4.00 × 10^−7^	(4.03 ± 0.01) × 10^−7^	100.7	0.2
	1.00 × 10^−5^	(0.91 ± 0.02) × 10^−5^	91.0	2.1
black bean	-	-	-	-
	2.00 × 10^−9^	(2.02 ± 0.04) × 10^−9^	101.0	1.9
	4.00 × 10^−7^	(4.05 ± 0.06) × 10^−7^	101.2	1.4
	1.00 × 10^−5^	(1.01 ± 0.02) × 10^−5^	101.0	1.98
millet	-	-	-	-
	2.00 × 10^−9^	(2.01 ± 0.02) × 10^−9^	100.5	0.99
	4.00 × 10^−7^	(4.06 ± 0.02) × 10^−7^	101.5	0.49
	1.00 × 10^−5^	(0.98 ± 0.03) × 10^−5^	98.0	3.06

## Data Availability

Not applicable.

## Supplementary Materials

The following are available online https://www.mdpi.com/article/10.3390/molecules27134294/s1, Figure S1: 1H NMR spectra of Fmoc-Ala-OH (**a**) and P(Fmoc-Ala-OH) (**b**), Figure S2: 1H NMR spectra of Fmoc-Thr-OH (**a**) and P(Fmoc-Thr-OH) (**b**), Figure S3: Fluorescence spectra of Fmoc-Ala-OH (**a**) and Fmoc-Thr-OH (**b**) toward Cr_2_O_7_^2−^ with different concentrations in DMSO/EtOH solution. Inset: linear plots of their fluorescence intensity against the Cr_2_O_7_^2−^ concentration, Table S1: Polymerization of monomers Fmoc-Ala-OH and Fmoc-Thr-OH in different solvents.

## Sample Availability

Not available.
