# Supplementary material for: High-Quality Conjugated Polymers Achieving Ultra-Trace Detection of Cr2O72− in Agricultural Products
Source: Molecules. 2022 Jul 4;27(13):4294. doi: 10.3390/molecules27134294 (PMC9268218; doi:10.3390/molecules27134294)
Supplement: Supplementary file 1 [file molecules-27-04294-s001.zip › molecules-1779989-supplementary.pdf]

Supplementary Material

High-quality conjugated polymers achieving ultra-trace detection of $Cr_2O_7^{2-}$ in agricultural products

Hui Li[1‡], Fei Li[1‡], Fang Liu[1], Xiao Chen[2], Wenyuan Xu[2], Liang Shen[2], Jingkun Xu[3,4,*] , Rui Yang[5,*], Ge Zhang[2,*]

[1]School of Pharmacy, Jiangxi Science & Technology Normal University, Nanchang 330013, P. R. China

[2]School of Chemistry & Chemical Engineering, Jiangxi Science & Technology Normal University, Nanchang 330013, P. R. China

[3]Jiangxi Key Lab of Flexible Electronics, Flexible Electronics Innovation Institute, Jiangxi Science & Technology Normal University, Nanchang 330013, Jiangxi, P.R. China

[4]College of Chemistry and Molecular Engineering, Qingdao University of Science & Technology, Qingdao 266042, P. R. China

[5]School of Electronics and Information Engineering, Changshu Institute of Technology, Changshu, 215500, China

*Address correspondence to E-mail: zhangge20082006@126.com (G. Zhang).

xujingkun1971@yeah.net (J. Xu)

yangruithrive@163.com (R. Yang)

[‡]These authors contributed equally to this work.

Table S1 Polymerization of monomers Fmoc-Ala-OH and Fmoc-Thr-OH in different solvents.

Polymerization / Monomers	THF	DMF	DCM	BFEE
Fmoc-Ala-OH	×	×	×	√
Fmoc-Thr-OH	×	×	×	√

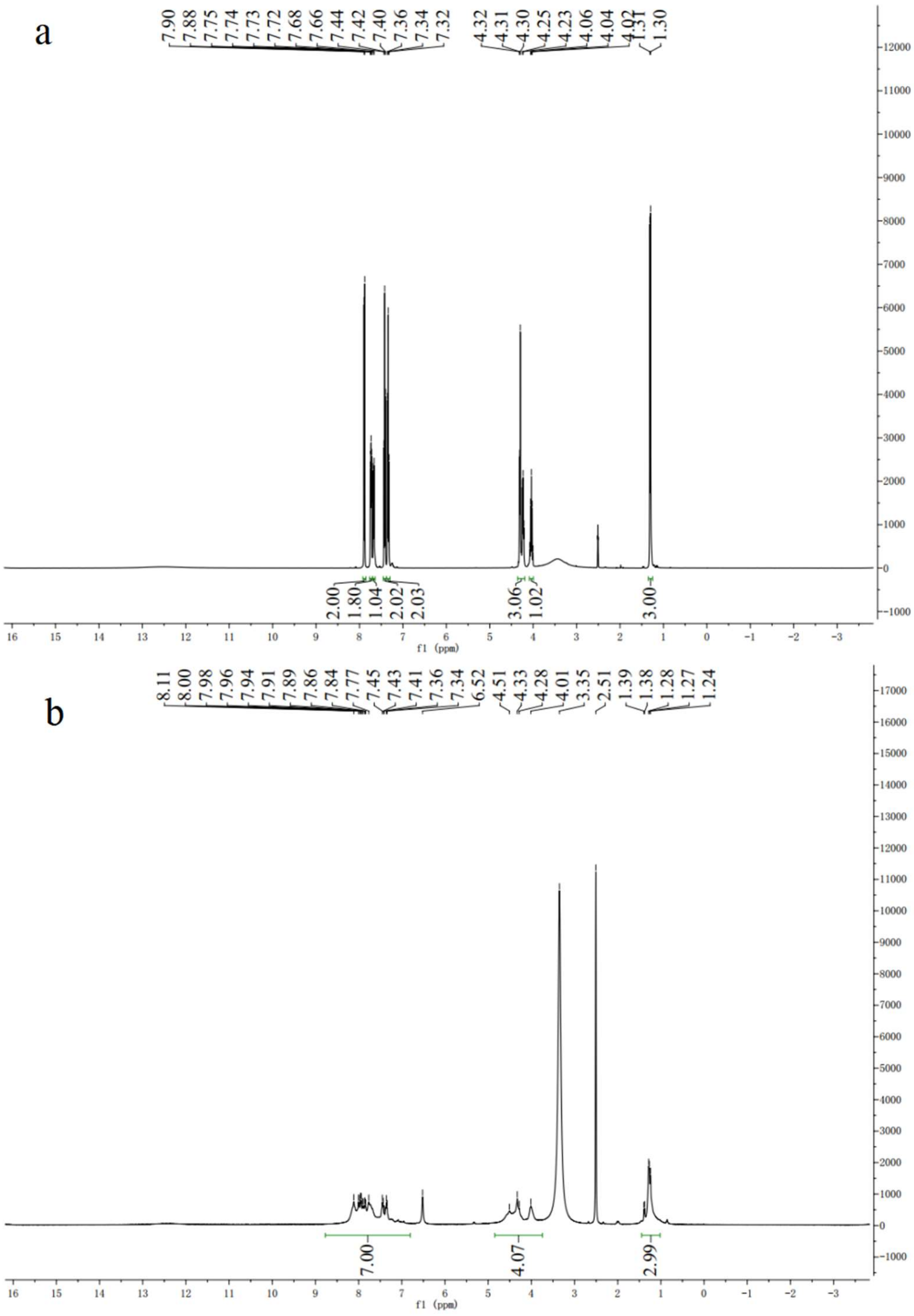

Figure S1. ^{1}H NMR spectra of Fmoc-Ala-OH (a) and **P(Fmoc-Ala-OH)** (b).

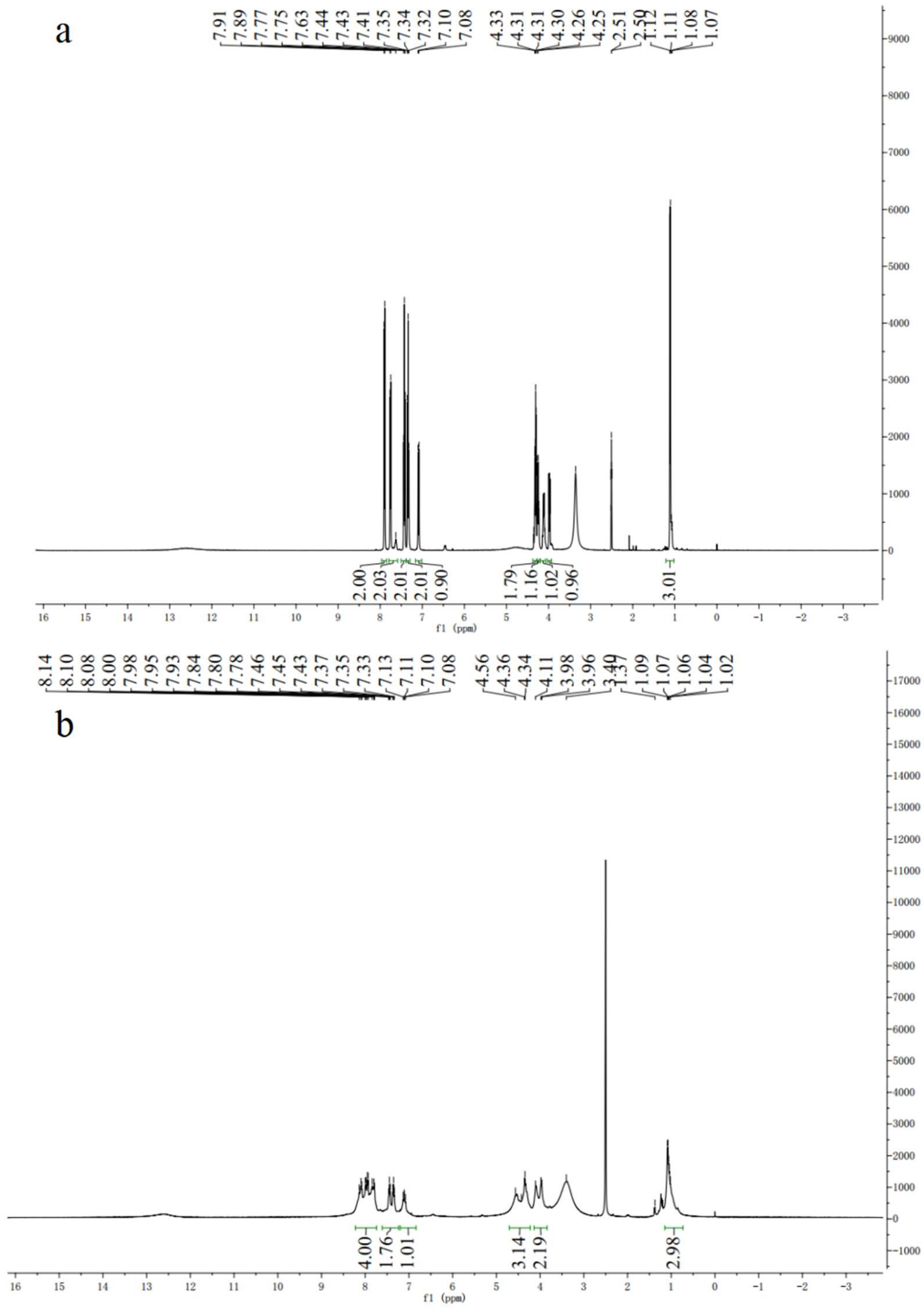

Figure S2. ^{1}H NMR spectra of Fmoc-Thr-OH (a) and **P(Fmoc-Thr-OH)** (b).

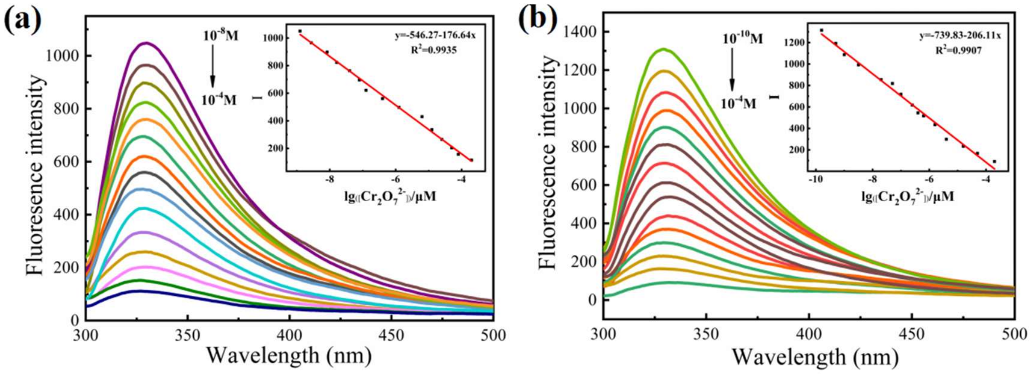

Figure S3. Fluorescence spectra of Fmoc-Ala-OH (a) and Fmoc-Thr-OH (b) toward $Cr_2O_7^{2-}$ with different concentrations in DMSO/EtOH solution. Inset: linear plots of their fluorescence intensity against the $Cr_2O_7^{2-}$ concentration.
